# Companion Animals Harbour Globally Circulating Human‐Associated *Klebsiella pneumoniae* Lineages and High‐Risk Antimicrobial Resistance Clones

**DOI:** 10.1155/tbed/5383720

**Published:** 2026-08-28

**Authors:** Stephen Mark Edward Fordham, Elizabeth Sheridan, Francis Drobniewski

**Affiliations:** ^1^ Department of Life and Environmental Sciences, Bournemouth University, Talbot Campus Fern Barrow, Poole BH12 5BB, UK, bournemouth.ac.uk; ^2^ Department of Medical Microbiology, Poole Hospital, University Hospitals Dorset NHS Foundation Trust, Longfleet Road, Poole BH15 2JB, UK; ^3^ Dept of Infectious Diseases, Imperial College London, 8th Floor, Office 8.N10, Hammersmith Campus, DuCane Road, London W12 ONN, UK, imperial.ac.uk

**Keywords:** companion animals, genomic surveillance, *Klebsiella pneumoniae*, multidrug resistance, One Health

## Abstract

Companion animals are increasingly recognised within One Health antimicrobial‐resistance (AMR) surveillance, yet the global genomic structure of dog‐ and cat‐associated *Klebsiella pneumoniae* remains poorly defined. We assembled a global dataset of 712 domestic dog (*Canis lupus familiaris*)– and cat (*Felis catus*)–derived *K. pneumoniae* genomes to define lineage diversity, AMR burden, host‐associated patterns, and overlap with human‐associated populations. Companion‐animal isolates comprised 263 sequence types (STs), but recurrent high‐risk lineages, including ST307, ST11, ST15, and ST147, were prominent. Among 706 isolates from 25 countries, extended‐spectrum β‐lactamase (ESBL) and carbapenemase genes were widely distributed, detected in 303 (42.9%) and 98 (13.9%) isolates, respectively. Cat‐derived isolates showed higher multidrug‐resistance (MDR) prevalence than dog‐derived isolates; 80.0% versus 56.3%, partly reflecting enrichment of epidemic clones, especially ST147. MDR was not confined to infection‐associated samples, indicating that colonisation may represent an important reservoir state. Comparison with 38,106 human‐associated *K. pneumoniae* isolates revealed extensive ST overlap, with 71.1% of companion‐animal STs and 87.2% of companion‐animal isolates belonging to STs also detected in humans. Focused recombination‐filtered phylogenomics of ST147 identified a closely related MDR cluster containing cat‐, dog‐ and human‐associated genomes, consistent with recent common ancestry and cross‐host cocirculation. Together, these findings reveal domestic dogs and cats harbour globally distributed *K. pneumoniae* lineages and high‐risk AMR clones also represented in human‐associated populations.

## 1. Introduction

Antimicrobial resistance (AMR) is a major global health challenge that requires coordinated surveillance across human, animal, and environmental sectors. The 2025 World Health Organisation (WHO) Global AMR and Use Surveillance System (GLASS) report highlights the scale of this threat, estimating that one in six laboratory‐confirmed bacterial infections caused by common bacterial pathogens in 2023 were resistant to antibiotic treatment [1]. Resistance among Gram‐negative pathogens is particularly concerning globally; more than 55% of *Klebsiella pneumoniae* (*K. pneumoniae*) infections were resistant to third‐generation cephalosporins (3GCs), a key treatment option for severe infections [1]. These trends reinforce the need for integrated One Health surveillance to identify reservoirs, track transmission pathways, and detect clinically important resistant lineages across connected human, animal, and environmental settings.

Within this One Health context, companion animals represent an increasingly important but comparatively undersurveilled population. Dogs and cats live in close contact with humans, share household environments, and may provide opportunities for bacterial exchange between people, animals, and shared surroundings. The scale of this close contact is substantial: in the United Kingdom, around 62% of households own a pet, including an estimated 15.5 million dogs and 13 million cats; across Europe, there are ~90 million dogs and 108 million cats, while the United States has an estimated 87.3 million owned dogs and 76.3 million owned cats [2–4]. Despite this, companion‐animal AMR surveillance remains less developed than human, livestock, and food‐chain surveillance. In the United Kingdom, this gap is now being addressed through Veterinary Medicines Directorate–led initiatives, including a national pilot delivered by Scotland’s Rural College to collect faecal samples from healthy dogs and cats in households, veterinary practices, and rescue centres to establish baseline AMR levels and evaluate practical surveillance approaches [5]. This United Kingdom system will add to an existing international companion–animal AMR surveillance landscape that includes the China AMR Surveillance Network for Pets (CARPet), established in 2021 to monitor clinical bacterial pathogens from companion animals [6].

Companion animals are increasingly recognised as reservoirs of multidrug‐resistant Enterobacterales, including clinically important *K. pneumoniae* lineages that overlap with human‐associated populations. However, the available evidence remains largely based on localised studies, household investigations, outbreak reports, or isolate collections enriched for extended‐spectrum β‐lactamase (ESBL)‐, AmpC‐, or carbapenemase‐producing strains [7–11]. As a result, the global population structure and lineage diversity of companion‐animal–associated *K. pneumoniae* remain poorly understood. Whether these isolates represent distinct host‐associated populations or are drawn from the same globally circulating strain pool as human‐associated *K. pneumoniae* has yet to be determined.

*K. pneumoniae* is a high‐priority AMR pathogen responsible for substantial morbidity and mortality worldwide, particularly when resistant to 3GCs or carbapenems [12, 13]. International high‐risk clones including ST11, ST15, ST147, ST258, and ST307 have expanded across healthcare systems and are frequently associated with ESBL and carbapenemase genes [14–17]. Importantly, related *K. pneumoniae* strains and sequence types (STs) have also been identified in companion animals and humans, including evidence of strain sharing within households [8].

Here, a global genome dataset of *K. pneumoniae* from domestic dogs and cats was assembled to define lineage diversity in these companion animals, AMR burden, host structure, and relationship to human‐associated populations. The distribution of ESBL and carbapenemase determinants, geographic and host‐associated multidrug resistance (MDR) patterns, source‐associated resistance carriage, dominant STs, and high‐risk clones was investigated. Companion‐animal ST comparisons against a large recent human PathogenWatch population were additionally performed to contextualise whether dog‐ and cat‐associated lineages overlap with globally circulating human *K. pneumoniae* clones.

## 2. Methods

### 2.1. Genome Dataset Assembly and Metadata Curation

Publicly available *K. pneumoniae* genome records associated with companion animals were identified from the European Nucleotide Archive (ENA), National Center for Biotechnology Information (NCBI) BioSample records, and the NCBI Pathogen Detection database, up to May 27, 2026, using host‐ and source‐associated terms for domestic dogs and cats. Metadata fields, including BioSample title, geographic location, collection date, strain name, and isolate identifiers, were standardised and merged into Supporting Information 1: File S1. Host metadata were standardised to dog or cat using common and scientific names, including *Canis lupus familiaris* and *Felis catus*. Records with human‐associated metadata, food or environmental sources, or unresolved companion–animal host assignment were excluded from host‐stratified analyses. Geographic metadata were standardised at country level (Supporting Information 1: File S1).

### 2.2. Genome Quality Control and Species Confirmation

Where read‐based assembly was required, draft genomes were generated using Shovill v1.1.0 [18] with a target depth of 100, minimum contig length of 500 bp, and minimum contig coverage of 5. Assembly quality was assessed from total assembly size, contig count, N50, and longest contig length. Assemblies were excluded if they were empty or outside quality thresholds: total size < 4.5 Mb or >7.0 Mb, >500 contigs, N50 < 20 kb, or longest contig < 50 kb. Species confirmation and downstream genotyping were performed using Kleborate v3.2.4 [19]; population analyses were restricted to isolates confirmed as *K. pneumoniae* from domestic dogs or cats.

### 2.3. MLST, AMR, and Virulence Gene Detection

ST, AMR determinants, resistance class counts, virulence loci, capsule, and O‐locus calls were extracted from Kleborate annotations [19]. ESBL and carbapenemase carriage were defined from Kleborate β‐lactamase fields, including Bla_ESBL_acquired and Bla_Carb_acquired. MDR was defined genotypically as resistance determinants spanning at least three antimicrobial classes; extensively drug‐resistant (XDR) isolates were included within the MDR/XDR category. Epidemic/high‐risk clone status was assigned to ST11, ST15, ST147, ST307, ST258, ST512, ST101, ST14, ST23, ST37, and ST392.

### 2.4. Geographic, Host, and Source‐Stratified Analyses

Country‐ and region‐level summaries were generated for isolate counts, ST composition, MDR/XDR burden, ESBL carriage, and carbapenemase carriage. Isolation sources were grouped as infection‐associated, colonisation/screening‐associated, or unknown using local and NCBI metadata. Infection‐associated terms included urine, bladder, blood, wound, abscess, eye, lung, clinical, diseased, and infection; colonisation/screening terms included faeces/feces, rectal, stool, perianal, and screening.

### 2.5. Rarefaction‐Based Comparison of Geographic AMR Patterns

To reduce the influence of uneven sampling intensity on geographic AMR comparisons, country‐ and region‐level analyses were supplemented with rarefaction. For country‐level comparisons, reporting countries/regions represented by at least 10 isolates were randomly subsampled to 10 isolates without replacement across 10,000 iterations. For each iteration, MDR/XDR proportion, ESBL/carbapenemase proportion, epidemic‐clone proportion, ST richness, Shannon diversity, and Simpson diversity were calculated; means and empirical 95% intervals were then summarised across iterations. Countries represented by fewer than 10 isolates were retained in descriptive summaries but excluded from rarefied country‐level comparisons.

### 2.6. Lineage Diversity and Statistical Analysis

ST richness, Shannon diversity, and Simpson diversity (1−D) were calculated from MLST profile counts. Dog richness was rarefied to the cat sample size by 9999 subsamples without replacement; 95% intervals were the 2.5th and 97.5th percentiles. Host differences in Shannon and Simpson diversity were tested using 9999 two‐sided label permutations. ST composition was compared by PERMANOVA using categorical dissimilarity (0 = same ST; 1 = different ST), with pseudo‐*F*, *R* ^2^, and *p*‐values derived from 9999 host‐label permutations and plus‐one correction. Country‐level associations between MDR/XDR burden and lineage diversity were assessed using Spearman correlation for countries with ≥10 isolates; geographic rarefaction used 10,000 iterations at a common depth of 10. Binary comparisons used Fisher’s exact tests, regional comparisons used chi‐square tests, and cat‐associated MDR/XDR was assessed by binomial logistic regression (maximum 200 iterations), with adjustment for clone status, country, year, and source. Human–animal ST similarity used Spearman correlation, Bray–Curtis dissimilarity, Jaccard overlap, and 10,000 permutations. Benjamini–Hochberg correction defined significance at *q* < 0.05. Analyses used Python 3.11.15, pandas 3.0.2, NumPy 2.4.3, SciPy 1.17.1, and statsmodels 0.14.6.

Human‐associated *K. pneumoniae* was incorporated through two distinct comparator designs. A metadata‐only PathogenWatch/ENA collection was used to assess population‐level MLST‐profile overlap, whereas lineage‐matched human ST147 genomes from NCBI Pathogen Detection cluster PDS000180176.25 were included for cluster‐focused phylogenomics. The latter dataset was not designed to estimate population prevalence, transmission probability, or transmission direction.

### 2.7. ST147 Comparator Genome Selection and Recombination‐Filtered Phylogenomics

ST147 phylogenomics used companion‐animal ST147 genomes together with contextual human and animal comparator genomes to construct a recombination‐filtered phylogeny for feline isolates detected in 2025–6. A lineage‐matched internal reference was selected using Mash v2.3 [20] by identifying the genome with the lowest mean pairwise distance. Core‐genome alignments were generated with Parsnp v2.1.5 and converted with harvesttools v1.3, both from the Harvest suite [21]. Recombinant regions were identified and masked using Gubbins v3.4.2 [22]. Maximum‐likelihood phylogenies were inferred from recombination‐filtered polymorphic sites using IQ‐TREE v3.1.1 under a GTR + G model with 1000 ultrafast bootstrap replicates [23]. Pairwise recombination–filtered SNP distances were calculated using snp‐dists v1.2.0.

### 2.8. Plasmid Replicon Profiling

Plasmid replicon profiles were assigned using BLASTN v2.12.0+ against a local PlasmidFinder replicon database [24]. Replicons were considered present using thresholds of ≥95% nucleotide identity and ≥60% replicon coverage. Replicon profiles were interpreted alongside core‐genome phylogeny, host category, collection year, and AMR genotype to assess accessory‐genome conservation within the ST147 lineage.

### 2.9. Construction of a Global Human *K. pneumoniae* Comparator Dataset

A contemporary metadata–only human comparator was assembled from public PathogenWatch records assigned to *K. pneumoniae* and retrieved on 01/03/2026. Isolate identifier, MLST profile, collection date, and geographic location were obtained through the PathogenWatch application programming interface (API); genome sequences were not downloaded. PathogenWatch BioSample accessions were queried against the ENA portal API to obtain host and isolation‐source metadata, and records were merged by BioSample accession. Isolates were retained when host or source annotations identified a human origin (*Homo sapiens*, human, or patient), collection year was 2019 or later, and a valid MLST assignment was available. ST labels were standardised for case and the ST prefix, while locus‐variant profiles were retained as distinct assignments rather than collapsed to canonical STs. Country was extracted from geographic‐location metadata, subnational information was removed, and country aliases were harmonised against ISO 3166‐1. Kosovo was retained as a named non‐ISO entity. The final comparator comprised 38,106 isolates spanning 1661 MLST profiles; 38,028 isolates had usable geographic metadata, representing 88 countries/territories. The curated metadata table and acquisition workflow were archived at https://github.com/StephenFordham/-Human_K.-pneumoniae_Comparator_Dataset_2019_onwards_SF/blob/main/humandataset_country_harmonised.csv.

## 3. Results

### 3.1. Host‐Shared Dominant Lineages Show Distinct Dog and Cat Enrichment

**Figure 1:**
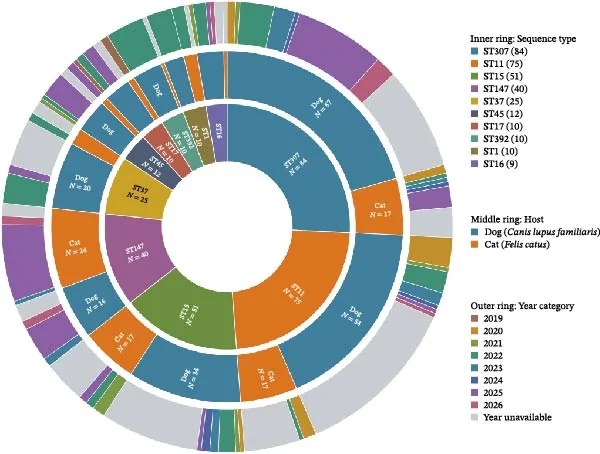
Host and temporal structure of dominant companion‐animal–associated *K. pneumoniae* lineages. Circular ring count plot shows the 10 most frequent STs among domestic dog‐ and cat‐derived *K. pneumoniae*. The inner ring shows the relative abundance of each ST, the middle ring partitions each ST by host species, and the outer ring shows collection‐year categories, including isolates without available year metadata. The dominant lineages were shared across host species but showed clear differences in host representation. ST307, ST11, and ST15 were the most abundant STs overall and were predominantly dog‐associated, whereas ST147 showed a comparatively stronger contribution from cat‐derived isolates while also including dog‐derived genomes. The year‐stratified outer ring indicates that several major STs were detected across multiple collection years, supporting recurrent recovery of these lineages from companion animals rather than isolated single‐time‐point observations. This structure highlights the coexistence of broadly distributed dog‐associated lineages and cat‐enriched high‐risk lineages within the companion‐animal *K. pneumoniae* population.

Across 712 domestic dog‐ and cat‐derived *K. pneumoniae* isolates, 263 STs were detected, indicating substantial lineage diversity in the companion‐animal population (Supporting Information 1: File S1). Despite this diversity, the ten most frequent STs accounted for 326/712 isolates (45.8%), comprising ST307 (*n* = 84), ST11 (*n* = 75), ST15 (*n* = 51), ST147 (*n* = 40), ST37 (*n* = 25), ST45 (*n* = 12), ST17 (*n* = 10), ST392 (*n* = 10), ST1 (*n* = 10), and ST16 (*n* = 9). All ten STs were detected in both dogs and cats, supporting host sharing rather than host restriction (Figure 1). However, their contribution differed by host: top‐10 STs accounted for 236/567 dog isolates (41.6%) but 90/145 cat isolates (62.1%), indicating that cat‐derived isolates were more concentrated within dominant lineages. The three most common STs, ST307, ST11, and ST15, represented 210/712 isolates (29.5%) and were predominantly dog‐associated, although each was also recovered from cats. In contrast, *K. pneumoniae* ST147 was the only top 10‐ST detected more often in cats (24 versus 16), reinforcing its cat‐enriched structure within an otherwise dog‐heavy population (Figure 1). Temporal metadata showed repeated detection of several major STs across recent years, with ST307 and ST11 represented from 2020 to 2026, ST15 from 2020 to 2025, and ST147 in 2023, 2025, and 2026 (Figure 1).

### 3.2. Comparison of Companion‐Animal and Human *Klebsiella pneumoniae* ST Distributions

To contextualise companion‐animal–associated *K. pneumoniae* within global human‐associated diversity, the domestic dog and cat dataset was compared with a large human comparator dataset described in Section 2. This comparator included 38,106 human‐associated *K. pneumoniae* isolates with ST assignments, collected from 2019 onwards across 88 countries and spanning 1661 STs. By comparison, the companion‐animal dataset comprised 712 isolates spanning 263 MLST profiles. ST sharing was extensive: 187/263 companion‐animal MLST profiles (71.1%) were also present in the human comparator, and 621/712 companion‐animal isolates (87.2%) belonged to ST profiles detected among human‐associated *K. pneumoniae*. All 10 most frequent companion‐animal STs were present in the human dataset, 19/20 of the top companion‐animal STs were detected in humans, and 10/20 were also among the 20 most frequent human STs. This overlap included several internationally recognised high‐risk clones, including ST11, ST15, ST17, ST147, ST307, ST258, and ST101.

**Figure 2:**
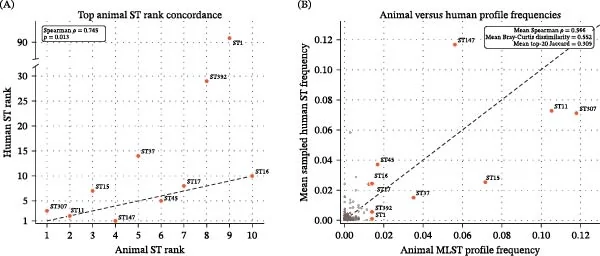
Concordance between companion‐animal–associated and human‐associated *K. pneumoniae* sequence type distributions. (A) Rank concordance between the 10 most frequent domestic dog/cat–associated *K. pneumoniae* STs and their corresponding ranks in the human comparator dataset. ST1 is shown using a broken *y*‐axis because it was common in the companion‐animal dataset but comparatively uncommon among human‐associated isolates. Rank concordance was significant across the top 10 companion‐animal STs (Spearman *ρ* = 0.745, *p* = 0.013). (B) Scatter plot comparing companion‐animal MLST‐profile frequency with mean sampled human ST frequency. Human frequencies were estimated from 100 random samples of 10,000 human‐associated *K. pneumoniae* isolates collected from 2019 onwards. The 10 most frequent companion–animal STs are highlighted in red; all other MLST profiles are shown in brown. Across sampling iterations, companion‐animal and human distributions showed moderate similarity among shared profiles (mean Spearman *ρ* = 0.566), with incomplete but nonrandom overlap in overall frequency structure (mean Bray–Curtis dissimilarity = 0.552; mean top‐20 Jaccard index = 0.309).

The overlap in ST composition was also reflected in lineage rank. Among the 10 most frequent companion‐animal STs, rank order was positively correlated with that observed in the human *K. pneumoniae* population (Spearman *ρ* = 0.745, *p* = 0.013; Figure 2A). Across 100 random samples of 10,000 human isolates, companion‐animal and human ST frequencies showed a moderate positive correlation among shared STs (mean Spearman *ρ* = 0.566), with a mean Bray–Curtis dissimilarity of 0.552 and a mean top‐20 Jaccard overlap of 0.309. A permutation test confirmed that the observed animal–human ST similarity was substantially greater than expected by chance (observed Bray–Curtis similarity = 0.448 versus null mean = 0.054; *p* = 0.00010, Figure 2B). Major shared lineages were represented contemporaneously in both datasets, although collection‐year overlap alone does not establish epidemiological linkage.

High‐risk clone burden was also comparable between populations using the high‐risk ST list (see Section 2): 262/712 companion‐animal isolates (36.8%) and 15,061/38,106 human isolates (39.5%) belonged to these clones (Fisher exact OR = 0.89, *p* = 0.142), indicating that companion animals share a similar proportion of clinically important lineages relative to the human *K. pneumoniae* population.

Temporal metadata additionally supported contemporary overlap for major shared lineages. Among the top 10 companion‐animal STs, five had animal records dated between 2019 and 2026 (ST307, ST11, ST15, ST147, and ST17), and all five were also detected among human‐associated *K. pneumoniae* in overlapping collection years. ST307 was detected in both datasets in 2022, 2024, and 2025; ST11 in 2023–2025; ST15 in 2023 and 2025; ST147 in 2023, 2025, and 2026; and ST17 in 2019 and 2025. At this population‐genetic scale, the extensive sharing of STs and ST‐associated profiles demonstrates substantial overlap between companion‐animal and human‐associated *K. pneumoniae* populations. This pattern is consistent with cocirculation of common lineages but does not identify individual transmission events or their direction.

### 3.3. Global Dissemination of ESBL and Carbapenemase Resistance Determinants in Companion Animal–Associated *K. pneumoniae*

To determine whether clinically important β‐lactam resistance determinants are disseminated among companion‐animal–associated *K. pneumoniae*, we examined ESBL and carbapenemase gene carriage among *K. pneumoniae* isolates recovered from domestic dogs and cats with available country metadata (*n* = 706). ESBL genes were common, being detected in 303/706 isolates (42.9%) and spanning 23 countries across four major geographic regions. Carbapenemase genes were less frequent but still widely distributed, occurring in 98/706 isolates (13.9%) across eight countries and three regions. In total, 345/706 isolates (48.9%) carried either an ESBL or carbapenemase gene, indicating that high‐priority β‐lactam resistance determinants are widespread in the companion animal collection (Supporting Information 1: File S1).

The burden of ESBL/carbapenemase carriage varied substantially by geography. The largest absolute numbers of ESBL/carbapenemase‐positive isolates were identified in the United States (96/231, 41.6%), France (71/108, 65.7%), China (65/124, 52.4%), South Korea (26/29, 89.7%), Switzerland (12/14, 85.7%), Brazil (11/12, 91.7%), Germany (10/52, 19.2%), and Spain (10/14, 71.4%). At regional scale, Asia had the highest proportion of ESBL/carbapenemase‐positive isolates (95/157, 60.5%), followed by Europe (135/281, 48.0%), the Americas (112/259, 43.2%), and Africa (3/8, 37.5%); no ESBL or carbapenemase genes were detected in the single isolate from Oceania. Geographic region was significantly associated with ESBL/carbapenemase carriage (chi‐square (χ^2^) = 13.24, df = 4, *p* = 0.0102), although this finding should be interpreted in the context of uneven sampling intensity across countries and regions.

The dissemination of these resistance determinants was strongly linked to dominant *K. pneumoniae* lineages. ESBL/carbapenemase‐positive isolates were concentrated in several internationally recognised high‐risk STs, including ST307 (*n* = 80 carriers across 13 countries and three regions), ST15 (*n* = 49 across nine countries and three regions), ST11 (*n* = 42 across nine countries and three regions), and ST147 (*n* = 33 across five countries and three regions). This pattern indicates that global dissemination is associated with the expansion and geographic spread of successful resistant lineages.

*bla*_CTX-M−15_ was the dominant ESBL determinant identified, detected in 231 isolates across 22 countries, consistent with broad international dissemination of this ESBL determinant in domestic dog/cat animal–associated *K. pneumoniae*. Other ESBLs were less frequent but included *bla*_CTX-M−55_ (*n* = 19), *bla*_CTX-M−27_ (*n* = 16), *bla*_CTX-M−14_ (*n* = 13), and *bla*_CTX-M−3_ (*n* = 10). Among carbapenemases, *bla*_NDM−5_ was the most common (*n* = 48 isolates across four countries), followed by *bla*_OXA−48_ (*n* = 31 across four countries), *bla*_KPC−3_ (*n* = 6), *bla*_NDM−7_ (*n* = 6), *bla*_OXA−181_ (*n* = 3), *bla*_OXA−232_ (*n* = 3), and *bla*_KPC−2_ (*n* = 2). Together, these results demonstrate that ESBL‐producing companion‐animal–associated *K. pneumoniae* is globally disseminated, while carbapenemase‐producing isolates, although less prevalent, are also distributed internationally and are concentrated within high‐risk lineages. These findings support companion animals as a reservoir for globally important β‐lactam resistance determinants and highlight the need to include domestic dogs and cats in genomic surveillance frameworks for antimicrobial‐resistant *K. pneumoniae*.

As ESBL and carbapenemase genes represent only part of the wider resistance burden, we next assessed MDR/XDR structure across countries and regions to determine whether geographic variation reflected overall resistance load, lineage composition, or sampling intensity.

### 3.4. Geographic Variation in MDR Burden Reflects Regional Lineage Structure After Rarefaction Normalisation

**Figure 3:**
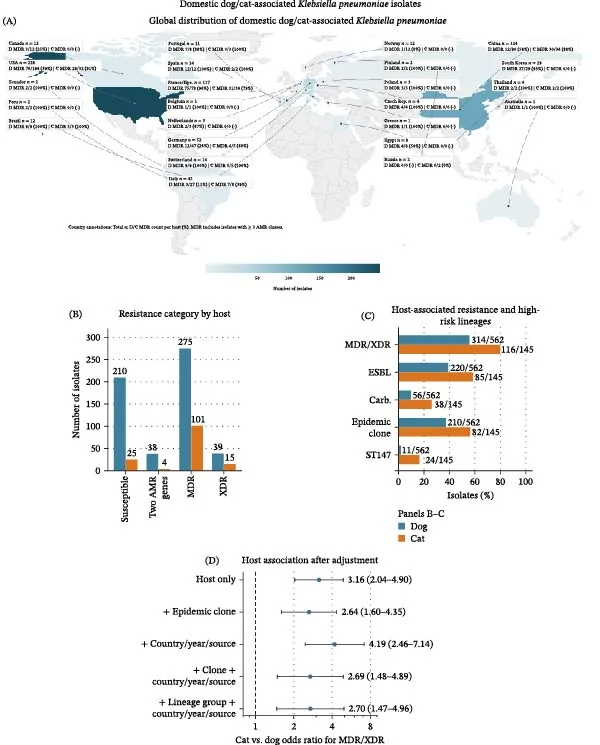
Global distribution, resistance burden, and host‐associated risk structure of domestic dog‐ and cat‐associated *K. pneumoniae*. 712 *K. pneumoniae* isolates were collated from domestic dogs (*n* = 567) and cats (*n* = 145). (A) Country‐level distribution of isolates; shading indicates the number of isolates per country, and annotations show total isolate counts and host‐stratified MDR burden. (B) Genotypic resistance categories among dog‐ and cat‐derived isolates, including susceptible, non‐MDR resistant, multidrug‐resistant (MDR), and extensively drug‐resistant (XDR) categories. (C) Host‐stratified prevalence of selected resistance and lineage features, including MDR/XDR genotype, ESBL genes, carbapenemase genes, epidemic clone membership, and ST147. (D) Logistic regression estimates for the association between cat origin and MDR/XDR carriage under sequential adjustment models. Points show cat‐versus‐dog odds ratios, and horizontal lines show 95% confidence intervals. MDR was defined as resistance determinants spanning three or more antimicrobial classes.

Geographic MDR burden and lineage diversity were assessed using dog/cat‐derived *K. pneumoniae* isolates with country metadata (*n* = 706). MDR/XDR was defined as carriage of resistance determinants spanning ≥ 3 antimicrobial classes. Observed absolute MDR/XDR counts were highest in the largest sampled settings, including France/Guadeloupe (106/117, 90.6%), the United States (110/231, 47.6%), China (82/124, 66.1%), South Korea (27/29, 93.1%), and Germany (16/52, 30.8%) (Figure 3A). As country sample sizes varied substantially, country‐level AMR burden was additionally normalised by rarefaction. Among the 12 reporting countries/regions represented by at least 10 isolates, repeated subsampling to 10 isolates confirmed persistently high MDR/XDR burden in Brazil, Switzerland, and Spain (mean rarefied MDR/XDR proportion 100% for each), South Korea (93.1%), Portugal (90.9%), and France/Guadeloupe (90.5%). Intermediate rarefied MDR/XDR burden was observed in China (66.3%) and the United States (47.2%), whereas Germany, Canada, Italy, and Norway showed lower rarefied estimates.

Regional analyses additionally supported geographic heterogeneity after accounting for unequal sampling. Observed regional sample sizes were 281 isolates from Europe, 259 from the Americas, 157 from Asia, eight from Africa, and one from Oceania. As Africa and Oceania were underpowered for rarefied inference, formal regional rarefaction was restricted to Asia, Europe, and the Americas at a common depth of 157 isolates. Rarefied MDR/XDR burden remained highest in Asia (72.0%), followed by Europe (65.5%) and the Americas (49.8%); rarefied ESBL/carbapenemase carriage showed the same ordering. Regional lineage structure differed substantially: Europe was enriched for recognised epidemic clones, particularly ST11, ST15, and ST307, with ST11 significantly enriched in Europe (OR = 9.95, BH‐adjusted *q* = 6.15 × 10^−15^). Asia was enriched for ST392 and ST37, whereas the Americas were enriched for ST147.

Rarefaction‐normalised country‐level analyses linked MDR/XDR burden to lineage concentration rather than sampling volume alone. Across the 12 country groups included in *n* = 10 rarefaction, mean rarefied MDR/XDR proportion was negatively correlated with rarefied ST richness, Shannon diversity, and Simpson diversity (Spearman rho = −0.859 for each; *p* = 0.000343) and positively correlated with rarefied epidemic‐clone proportion (rho = 0.931; *p* = 1.08 × 10^−5^). These findings indicate that geographic differences in MDR/XDR burden are shaped by regional lineage structure and epidemic‐clone enrichment, while rarefaction reduces the risk that cross‐country comparisons simply reflect unequal sampling intensity.

### 3.5. Domestic Dogs Harbour Broader and More Even *K. pneumoniae* ST Diversity Than Cats

The geographic analyses indicated that resistance burden was closely linked to lineage structure; therefore, we next compared ST diversity directly between dog‐ and cat‐derived isolates to determine whether host species differed in the breadth and evenness of their *K. pneumoniae* populations. Among *K. pneumoniae* isolates with ST assignment calls belonging to either domestic dogs or cats (*n* = 712; dogs *n* = 567, cats *n* = 145), dogs harboured substantially more diverse ST lineages than cats. Dogs had higher observed ST richness (240 STs vs. 51 in cats), higher Shannon diversity (*H* = 4.569 vs. 3.187; permutation test, delta *H* = 1.382, *p* = 0.0001, 9999 permutations), and lower lineage dominance/higher Simpson diversity (1‐D = 0.967 vs. 0.924; permutation *p* = 0.0004). As sampling was unequal, richness was rarefied to the cat sample size (*n* = 145). Here, dogs still had higher expected ST richness (84.08 STs; Monte Carlo 95% interval 75–93) than cats (51 STs), with a rarefied richness difference of 33.08 STs (*p* = 0.0001). PERMANOVA based on categorical ST dissimilarity showed that host species was also associated with a statistically significant difference in lineage composition (pseudo‐*F* = 3.357, *R* ^2^ = 0.0047, *p* = 0.0001, 9999 permutations), although the low *R* ^2^ indicates that host explains only a small fraction of total ST compositional variation. Overall, the data support that dog‐derived isolates contain a broader and more even set of *K. pneumoniae* ST lineages than cat‐derived isolates.

### 3.6. Higher MDR/XDR Prevalence Among Cat‐Derived Isolates Is Partly Explained by High‐Risk Lineage Structure

Cat‐derived *K. pneumoniae* isolates showed a higher MDR/XDR prevalence than dog‐derived isolates (116/145, 80.0% versus 319/567, 56.3%; Fisher exact OR = 3.11, 95% CI 2.00–4.83; *p* = 7.29 × 10^−8^). Cat isolates also more frequently carried ESBL genes (58.6% versus 39.2%; OR = 2.20, 95% CI 1.52–3.19; *p* = 3.23 × 10^−5^) and carbapenemase genes (26.2% versus 10.8%; OR = 2.95, 95% CI 1.87–4.65; *p* = 6.57 × 10^−6^) (Figure 3B,C). This indicates that the higher MDR/XDR burden in cats coincided with enrichment for clinically important β‐lactam resistance determinants.

This host‐associated pattern was partly explained by lineage structure. Cat isolates were more likely than dog isolates to belong to recognised epidemic/high‐risk clones (ST11, ST15, ST147, ST307, ST258, ST512, ST101, ST14, ST23, ST37, and ST392) (56.6% versus 37.9%; OR = 2.13, 95% CI 1.47–3.08; *p* = 6.78 × 10^−5^). ST147 was also strongly over‐represented in cats (16.6% versus 2.8%; OR = 6.83, 95% CI 3.52–13.25; *p* = 1.62 × 10^−8^) (Figure 3C). In logistic regression, cat origin was associated with MDR/XDR carriage in a host‐only model (OR = 3.11, 95% CI 2.00–4.83; *p* = 4.19 × 10^−7^) (Figure 3D). Adjustment for epidemic/high‐risk clone status reduced, but did not remove, this association (OR = 2.63, 95% CI 1.60–4.34; *p* = 1.51 × 10^−4^), while epidemic‐clone carriage itself was strongly associated with MDR/XDR (OR = 18.01, 95% CI 11.23–28.87; *p* = 3.51 × 10^−33^).

Country, year, and source did not explain away the cat‐associated MDR/XDR signal. Adjustment for country, collection year, and source category gave a cat‐associated OR of 4.07 (95% CI 2.39–6.92; *p* = 2.37 × 10^−7^), while a model additionally including epidemic‐clone status gave an OR of 2.67 (95% CI 1.47–4.86; *p* = 0.00128) (Figure 3D). A reduced lineage model separating ST147, other epidemic clones, and nonepidemic lineages, while also adjusting for country, year, and source, gave a similar cat effect (OR = 2.63, 95% CI 1.43–4.82; *p* = 0.00178). As full ST fixed–effect models were unstable in this dataset due to rare STs and near‐complete MDR/XDR in several lineages, ST‐aware inference was also assessed using a within‐ST permutation test restricted to STs present in both hosts. This retained a host‐associated difference in MDR/XDR carriage (28 ST strata; 399 isolates; observed cat‐minus‐dog difference = 7.3 percentage points; 99,999 permutations, *p* = 0.0197).

Overall, cat‐derived isolates had higher MDR/XDR prevalence than dog‐derived isolates, and this association persisted after adjustment for measured country, year, source, and broad lineage structure. However, the effect was attenuated after accounting for epidemic/high‐risk clones, indicating that clonal composition contributes to the host difference. These results should therefore be interpreted as an epidemiological association rather than evidence of host‐driven causation. Higher MDR/XDR prevalence among cat‐derived isolates may also reflect a combination of high‐risk clone expansion, sampling framework, geography, veterinary healthcare exposure, antimicrobial use history, and host‐associated epidemiology, rather than cats being more likely to harbour MDR *K. pneumoniae*.

### 3.7. MDR *K. pneumoniae* Are Not Restricted to Infection‐Associated Samples

MDR carriage was not confined to infection‐associated isolates. MDR was detected in 82/132 colonisation/screening‐associated isolates (62.1%) compared with 193/368 infection‐associated isolates (52.4%). Although the observed proportion was higher among colonisation/screening‐associated isolates, this difference did not reach statistical significance in the unadjusted comparison (Fisher exact test, OR = 0.67 for infection‐associated versus colonisation/screening‐associated isolates, 95% CI 0.45–1.01, *p* = 0.0663).

After adjustment for host species, the source‐associated difference was further attenuated and remained nonsignificant (adjusted OR = 0.82, 95% CI 0.53–1.26, *p* = 0.354), indicating that host composition partly explains the observed unadjusted pattern. These results indicate MDR *K. pneumoniae* are frequently recovered from colonisation or screening‐associated sources as well as infection‐associated sources. This supports the potential role of domestic dogs and cats as important reservoirs of MDR lineages beyond clinically diseased animals.

### 3.8. A Cat‐Enriched MDR ST147 Cluster Includes Closely Related Companion‐Animal and Human‐Associated Genomes

As ST147 was both cat‐enriched and strongly associated with clinically important resistance determinants, with strains recently detected in 2025 and 2026, we performed focused phylogenomic analysis to determine whether companion animal ST147 represented a host‐restricted expansion or part of a broader host‐spanning lineage.

Recombination‐aware phylogenomic analysis placed the companion‐animal ST147 *K. pneumoniae* isolates within a structured and heterogeneous ST147 population, rather than within a single animal–restricted lineage. Here, a cat‐enriched sublineage was identified. This lineage, however, was not exclusively feline: Dog‐derived isolates, additional animal‐context isolates, and closely related human comparator genomes were embedded within or immediately adjacent to this sublineage. In contrast, other cat‐ and dog‐associated ST147 genomes occupied more distant positions in the tree, revealing companion‐animal isolates were distributed across multiple phylogenetically distinct ST147 backgrounds rather than forming a single animal–associated cluster.

**Figure 4:**
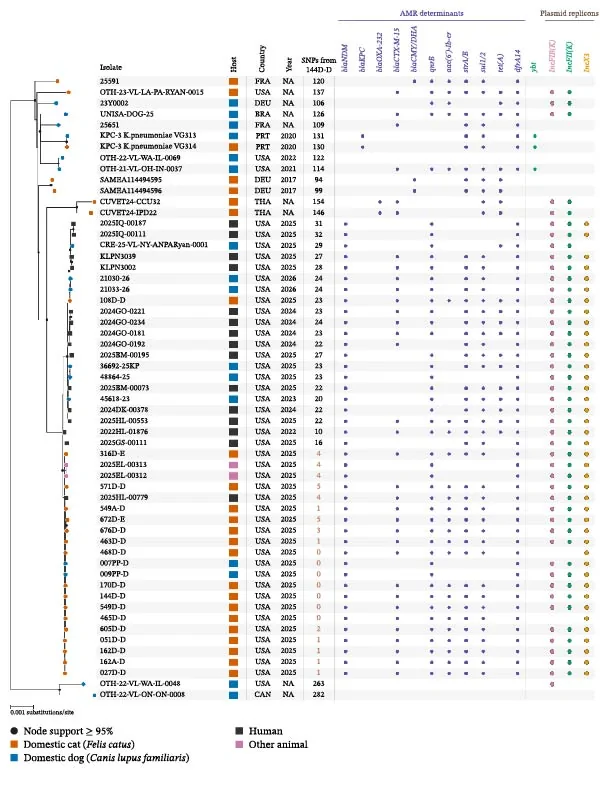
Recombination‐filtered core‐genome phylogeny of ST147 *K. pneumoniae* isolates from companion animals and contextual human genomes. The maximum‐likelihood tree comprises 57 genomes, including all isolates in NCBI Pathogen Detection cluster PDS000180176.25 and additional dog‐ and cat‐derived ST147 genomes identified in the study dataset. Assemblies were aligned with Parsnp; recombinant regions were masked with Gubbins, leaving 1054 polymorphic sites for IQ‐TREE inference. Filled internal nodes indicate bootstrap support ≥ 95%, and the scale bar denotes substitutions per site. Tip colour identifies host: domestic cat (orange), domestic dog (blue), human (black), or other animal (pink); human isolates are shown as squares and animal isolates as circles. Adjacent columns report ISO 3166‐1 alpha‐3 country codes, collection year, and recombination‐filtered SNP distance from USA‐NY cat isolate 144D‐D (SAMN48918375), with values ≤ 5 SNPs emphasised. Unavailable country or year metadata are marked NA. Filled circles denote detection of selected acquired antimicrobial‐resistance determinants, yersiniabactin (ybt), and IncFIB(K), IncFII(K), or IncX3 plasmid replicons; blank cells indicate nondetection.

The feline‐dominant lineage showed limited core‐genome diversity. Among the 25 cat‐ and dog‐derived isolates in this lineage, pairwise recombination–filtered distances ranged from 0 to 34 SNPs, with a median of 6 SNPs. The 17 USA‐NY feline isolates formed a tight cluster, differing by 0–28 SNPs, with a median of 3 SNPs. Using the USA‐NY cat isolate 144D‐D (SAMN48918375) as a fixed feline–clade comparator, several cat isolates and both USA‐NY dog isolates were 0 SNPs from this genome, while most remaining USA‐NY cat isolates were within 1–5 SNPs (Figure 4). These distances are consistent with recent diversification of a highly related ST147 sublineage represented in both companion‐animal hosts.

Human comparator genomes were closely associated with the animal‐associated cluster. The nearest human genome to each animal isolate, within the feline‐dominated sublineage, was separated by only 2–9 recombination‐filtered SNPs; across all animal‐to‐human comparisons within the NY feline–dominated lineage, distances ranged from 2 to 37 SNPs, with a median of 22 SNPs. Several USA‐NY cat isolates were 3–4 SNPs from human isolate 2025HL‐00779, and dog‐derived isolate 45618‐23 was 2 SNPs from human isolate 2024DK‐00378. These data demonstrate close genomic relatedness and recent common ancestry among ST147 genomes sampled from animal and human sources. However, in the absence of epidemiological linkage, these phylogenetic relationships should be interpreted as evidence of a shared circulating background rather than proof of direct transmission. The distinctiveness of the focal lineage was further supported by comparison with ST147 genomes outside this sublineage: Distances from the animal isolates within the cat‐dominated sublineage to external ST147 comparators were substantially larger, ranging from 93 to 298 SNPs, with a median of 130 SNPs (Figure 4).

The host distribution, resistance profile, and accessory‐genome structure together define a cat‐enriched, MDR ST147 sublineage that is not host restricted. Within the compact animal–associated lineage, all 25 cat‐ and dog‐derived isolates carried the same core resistance variants, comprising the carbapenemase gene *bla*_NDM−5_, the quinolone‐resistance gene *qnrB1*, and the trimethoprim‐resistance gene *dfrA14*, indicating a conserved MDR genotype across the companion‐animal component of the clade. However, the phylogeny does not support a strictly feline lineage: Dog‐derived isolates, animal‐context isolates, and closely related human comparator genomes were interspersed within, or positioned immediately adjacent to, the same genomic background (Figure 4). These results identify a compact MDR ST147 cluster enriched among cats and represented by cat‐, dog‐, and human‐associated genomes. The analysis establishes close genomic relatedness across host‐associated collections but not transmission between hosts. This is supported by the low recombination–filtered SNP distances within the feline‐dominated cluster and the close proximity of human comparator isolates.

Plasmid replicon profiling further showed that this lineage sits within a broader ST147 accessory–genome background dominated by recurrent Inc groups. Across the complete set of 57 ST147 genomes analysed by recombination‐filtered core‐genome phylogeny and plasmid replicon profiling, the most frequent replicon groups were IncFIB(K), IncFII(K), and IncX3, detected in 46/57, 45/57, and 41/57 genomes, respectively (Figure 4). These replicons were present across human, cat, dog‐derived, and animal‐context isolates and were retained across the feline‐dominated clade rather than being confined to a single host category. Among dated recurrent‐replicon–positive genomes, all three major replicons were already present in the 2022 human isolate 2022HL‐01876 (SAMN31854197), were subsequently detected in the 2023 dog‐derived isolate 45618‐23 (SAMN40717015), and persisted through the 2024–2026 expansion that included human comparators, the USA‐NY cat cluster, and later dog‐derived isolates sampled in 2025 and 2026. This temporal pattern supports persistence of a conserved IncFIB(K)/IncFII(K)/IncX3 plasmid–replicon architecture within the ST147 population before and during the observed companion‐animal expansion.

In contrast to these dominant recurring replicons, additional Inc groups were uncommon and generally restricted to one or two genomes, consistent with episodic plasmid gain, loss, or replacement against a stable accessory–genome backbone. The combined core–genome and plasmid‐replicon data therefore provide no evidence that the cat‐associated isolates carried a wholly distinct plasmid–replicon profile. Instead, the cat‐, dog‐, and human‐associated genomes represent closely related components of an MDR ST147 lineage sharing a recurrent plasmid–replicon repertoire. This cross‐host overlap does not establish movement of the lineage or plasmids between host populations. The convergence of low core–genome SNP distances, conserved MDR determinants, and recurrent plasmid replicons highlights the value of integrated human and companion‐animal genomic surveillance for detecting high‐risk *K. pneumoniae* lineages at the human–animal interface.

### 3.9. Host Comparison of ST147 Accessory Gene Content

To assess whether resistance‐associated accessory content was shared across hosts, acquired AMR genes, accessory virulence loci, and plasmid replicons were compared across 57 ST147 genomes (40 dog/cat, two other‐animal, and 15 human isolates). All 11 AMR genes detected in human isolates were also present in dog/cat isolates, whereas 11/39 (28.2%) genes detected in dog/cat isolates occurred in humans. Frequently shared determinants included *bla*_NDM−5_, *bla*_CTX-M−15_, *qnrB1*, *dfrA14*, *sul2*, *strA*, and *strB*, indicating substantial overlap in the dominant resistance repertoire, although animal isolates carried additional low‐frequency genes. IncFIB(K), IncFII(K), and IncX3 occurred in 72.5%, 70.0%, and 60.0% of dog/cat isolates, respectively, and in all human isolates. The combined profile occurred in 22/40 (55.0%) dog/cat and 15/15 human isolates. These findings support cross‐host overlap in major resistance determinants and plasmid‐replicon architecture but not complete plasmid identity, which requires confirmation using complete long‐read or hybrid assemblies. Full gene‐level frequencies are provided in Supporting Information 2: Table S1.

## 4. Discussion

Companion‐animal–associated *K. pneumoniae* comprises a diverse population containing globally distributed high‐risk STs also prominent in human‐associated collections. Extensive ST overlap supports contemporaneous cocirculation within a shared population background rather than distinct human and veterinary lineage pools. *K. pneumoniae* is a major contributor to the global burden of bacterial AMR and a highly plastic opportunistic pathogen with a well‐recognised capacity to acquire and disseminate AMR determinants across ecological niches [12, 25]. The recovery of these lineages from companion animals provides support that dogs and cats form part of the broader One Health ecology of *K. pneumoniae*. This is consistent with increasing evidence that AMR bacteria of human clinical relevance occur in companion animals, households, and veterinary settings [10].

Shared STs are low‐resolution indicators of relatedness and cannot establish direct transmission, epidemiological linkage, or directionality. Public genomic datasets also carry sampling biases related to geography, sequencing capacity, outbreak investigation, and preferential submission of resistant isolates, which may influence apparent lineage frequencies [26]. However, the scale and consistency of the overlap observed, together with the enrichment of recognised high‐risk clones, indicate that companion animals harbour *K. pneumoniae* lineages drawn from the same wider strain pool as human‐associated isolates.

The similar MDR rates in infection‐associated and colonisation/screening‐associated *K. pneumoniae* suggest that resistant strains are not found only in animals with clinical disease but can also be carried by animals without obvious infection. This is epidemiologically important because colonisation can provide a clinically silent reservoir from which later infection may arise. In humans, gastrointestinal carriage of *K. pneumoniae* is increasingly recognised as a precursor state for infection, with studies showing that colonising strains can subsequently cause clinical disease and that resistant *K. pneumoniae* may persist unnoticed in healthcare‐associated populations [27]. Comparable patterns have been reported in companion animals, where longitudinal studies have shown recurrent or persistent faecal carriage of ESBL/AmpC‐producing Enterobacterales in dogs, including postdischarge carriage with associated household environmental contamination [28, 29]. Household studies have also identified carriage, acquisition, and cocarriage of ESBL‐producing Enterobacterales among dogs, cats, and cohabiting humans [30]. Together, these findings indicate that colonisation‐associated companion‐animal isolates should be regarded as epidemiologically important rather than incidental, while direct transmission should not be inferred without paired owner–animal genomic sampling.

Although clonal composition was the strongest measurable explanation for higher MDR prevalence in cats, the factors shaping this lineage structure remain uncertain. Veterinary healthcare exposure, recurrent urinary disease, prior antimicrobial treatment, and repeated exposure to similar antimicrobial classes are recognised drivers of MDR Enterobacterales in companion animals [31, 32], but these metadata were unavailable for analysis here. Cat‐derived isolates from China were enriched for urinary sources, consistent with previous reports linking feline *K. pneumoniae* infection to urinary tract disease [33], although this pattern was not observed globally. These factors provide a clinical and sampling context for why feline isolates may be enriched for MDR‐associated lineages, but they cannot be directly tested without individual‐level treatment, hospitalisation, and disease‐history data. Public‐genome ascertainment bias may also contribute, as feline isolates are less frequently available and may be preferentially sequenced when clinically significant, unusual, or antimicrobial resistant. Overall, the data indicate that enrichment of ST147 and other epidemic clones is the main measurable explanation for the higher MDR prevalence in cats, while veterinary exposure, antimicrobial selection, urinary‐disease sampling, and public‐database ascertainment bias remain plausible but unproven contributors.

ST147 is a globally disseminated high‐risk *K. pneumoniae* clone associated with MDR, ESBLs, and carbapenemases, including NDM‐, OXA‐48‐like, and KPC‐type enzymes [16]. Within ST147, low recombination–filtered SNP distances and overlap in MDR determinants and IncFIB(K)/IncFII(K)/IncX3 replicons indicate recent common ancestry among genomes sampled from different hosts. However, incomplete sharing of accessory genes, replicon markers, and virulence loci does not support complete plasmid identity. These findings are compatible with cocirculation of a shared ST147 background but cannot distinguish direct cross‐species transmission from independent or indirect acquisition through unsampled reservoirs [17, 34].

Companion animals are exposed to antimicrobials that can select for MDR Enterobacterales [35, 36], potentially maintaining MDR *K. pneumoniae* once introduced into animal, household, or veterinary‐hospital environments. However, carbapenems are generally not licensed or routinely used in veterinary medicine; therefore, carbapenemase carriage in companion animals may likely reflect acquisition of resistant strains or mobile elements from human‐associated, environmental, or healthcare‐linked reservoirs rather than direct carbapenem selection [37]. Overall, the cat‐enriched ST147 sublineage does not provide evidence that cats are the source of this clone, but it shows that companion animals can carry closely related, clinically consequential MDR lineages overlapping with human‐associated genomic diversity.

Collectively, these findings indicate that companion animals should not be viewed as peripheral or epidemiologically separate from the wider *K. pneumoniae* AMR landscape. Instead, dogs and cats can harbour globally disseminated MDR lineages, including high‐risk clones with resistance profiles closely related to those observed in human‐associated populations. The data do not establish directionality, source attribution, or direct transmission, and interpretation is limited by uneven public‐genome sampling and incomplete clinical metadata. Nevertheless, the combination of ST overlap, MDR clone enrichment, colonisation‐associated recovery, and fine‐scale ST147 relatedness supports the inclusion of companion animals in targeted One Health genomic surveillance.

## Author Contributions

**Stephen Mark Edward Fordham**: conceptualisation (lead), writing – original draft (lead), formal analysis (lead), writing – review and editing (lead), visualisation (lead). **Elizabeth Sheridan**: conceptualisation (supporting), writing – review and editing (equal). **Francis Drobniewski**: conceptualisation (supporting), formal analysis (supporting), writing – review and editing (equal).

## Funding

No funds, grants, or other support were received for conducting this study or preparing this manuscript.

## Ethics Statement

Ethical approval was not required because this study used publicly available bacterial genome sequences and associated metadata and did not involve new sampling of animals or humans.

## Conflicts of Interest

The authors declare no conflicts of interest.

## Supporting Information

Additional supporting information can be found online in the Supporting Information section.

## Supporting information


**Supporting Information 1** File S1: Curated metadata, assembly‐quality metrics, and genomic characterisation of candidate dog‐ and cat‐associated *Klebsiella* genomes. The dataset contains 791 publicly available genome records identified during the assembly of the companion‐animal *Klebsiella pneumoniae* collection. For each record, it provides accession and isolate identifiers, original and standardised host and isolation‐source metadata, geographic and collection information, genome‐assembly quality metrics, species identification, multilocus sequence typing results, acquired antimicrobial‐resistance determinants, resistance‐class and gene counts, virulence‐associated loci, and capsule and O‐antigen locus assignments generated using Kleborate. The final analytical population comprised 712 *K. pneumoniae* genomes with resolved domestic dog or cat host assignments. Non‐*K. pneumoniae* records and records without sufficiently resolved host assignments are retained to document dataset screening and exclusion.

**Supporting Information 2** Table S1: Host‐stratified ST147 accessory resistance, virulence, and plasmid‐replicon content. Table S1: The frequency of acquired AMR genes, accessory virulence loci/genes, and PlasmidFinder replicon markers among 57 ST147 *K. pneumoniae genomes* used in the host‐comparison analysis. The dataset comprised 40 dog/cat isolates, two other‐animal isolates, and 15 human comparator isolates. For each feature, frequencies are shown separately for cat, dog, combined dog/cat, other‐animal, and human isolates. Fisher exact tests compare combined dog/cat isolates with human isolates; Benjamini–Hochberg *q*‐values are provided for descriptive multiple‐testing correction across tested features.

## Data Availability

The data that support the findings of this study are available from the corresponding author upon reasonable request.
